# The Role of BK Channels in Antiseizure Action of the CB1 Receptor Agonist ACEA in Maximal Electroshock and Pentylenetetrazole Models of Seizure in Mice

**Published:** 2017

**Authors:** Sina Asaadi, Mohammad Jahanbakhshi, Mahmoud Lotfinia, Nima Naderi

**Affiliations:** a *Neuroscience Research Center, Shahid Beheshti University of Medical Sciences, Tehran, Iran. *; b *School of Medicine, Shahid Beheshti University of Medical Sciences, Tehran, Iran.*; c *Department of Pharmacology and Toxicology, School of Pharmacy, Shahid Beheshti University of Medical Sciences, Tehran, Iran.*

**Keywords:** BK channel, Cannabinoid, Pentylenetetrazole, Maximal Electroshock, Seizure, Mice

## Abstract

The anticonvulsant effect of cannabinoid compound has been shown in various models of seizure. On the other hand, there are controversial findings about the role of large conductance calcium-activated potassium (BK) channels in the pathogenesis of epilepsy. Also, there is no data regarding the effect of co-administration of cannabinoid type 1 (CB1) receptor agonists and BK channels antagonists in the acute models of seizure in mice. In this study, the effect of arachidonyl-2′-chloroethylamide (ACEA), a CB1 receptor agonist, and a BK channel antagonist, paxilline, either alone or in combination was investigated. Both pentylenetetrazole (PTZ) and maximal electroshock (MES) acute models of seizure were used to evaluate the protective effects of drugs. Mice were randomly selected in different groups: (i) control group; (ii) groups that received different doses of either paxilline or ACEA; and (iii) groups that received combinations of ACEA and paxillin at different doses. In MES model, prevention of hindlimb tonic extension (HLTE) was considered as protective effect. In PTZ model, the required dose of PTZ (mg/kg) to induce tonic-clonic seizure with loss of righting reflex was considered as seizure threshold. In PTZ model, while administration of ACEA per se (5 and 10 mg/kg) caused protective effect against seizure; however, co-administration of ACEA and ineffective doses of paxilline attenuated the antiseizure effects of paxilline. In MES model, while pretreatment by ACEA showed protective effects against seizure; however, co-administration of paxilline and ACEA caused an antagonistic interaction for their antiseizure properties. Our results showed a protective effect of ACEA in both PTZ and MES acute models of seizure. This effect was attenuated by co-administration with paxilline, suggesting the involvement of BK channels in antiseizure activity of ACEA.

## Introduction

Exogenous cannabinoids and their endogenous congeners modulate excitability of nervous system and bursting activity of neurons through different mechanisms and produces a balance between excitatory and inhibitory neurotransmitters in the central nervous system (CNS) (1, 2) (3). The cannabinoid system has been demonstrated to play an important role in regulating seizure activity in the brain, which might offer them as a new adjuvant therapy in patients with severe or uncontrollable seizures (4). 

Large conductance calcium-activated potassium (BK) Channels are expressed throughout the CNS (5, 6). Under physiological conditions, activation of neuronal BK channels contributes to action potential repolarization, resulting in fast afterhyperpolarization (fAHP) following action potential. The action of BK channels changes the shape of dendritic calcium spikes and influence neurotransmitter release. Thus, activation of BK channels could be considered as an intrinsic inhibitory mechanism to oppose membrane depolarization and the excessive accumulation of cytosolic calcium that occurs during seizures (7-9). Increasing potassium efflux into the cytoplasm of neurons could stabilize resting membrane potential resulting in membrane hyperpolarization and reduced neural excitability (10). On the other hand, BK channels are possibly involved in absence seizure and temporal lobe epilepsy by narrowing action potentials and facilitate high-frequency firing (11, 12). Paxilline is a highly specific BK-channel antagonist that acts in the low nanomolar range (13), which could pass the blood–brain barrier (14). Because of this, paxilline was chosen as a potential therapeutic agent to decrease the effects of BK-channel gain-of-function in catalyzing abnormal activity and, potentially, seizure initiation (15).

The role of BK channels in anticonvulsant effects of CB1 receptor agonist in acute models of seizure is not completely clear. In this study, we sought to examine whether in vivo administration of a paxilline, a highly specific BK-channel antagonist, could change the anticonvulsant effect of arachidonyl-2′-chloroethylamide (ACEA), the highly selective CB1 receptor agonist, in pentylenetetrazole (PTZ) and maximal electroshock (MES) models.

## Materials and Methods

*Animals*

Male NMRI mice weighing 20–30 g (Pasture Institute, Tehran, Iran) were used in this study. Animals were kept in a room with 12 h/12 h light/dark cycle (lights on at 7:00 a.m.) and controlled temperature (22 ± 2 °C) with free access to food and tap water except in short time during experiments. Each animal was used only once. All experiments were performed between 1:00 p.m. and 6:00 p.m. All procedures were in accordance with the National Institute of Health Guide for the Care and Use of Laboratory Animals (NIH Publications No. 80-23, revised 1996) and were approved by the local Research and Medical Ethics Committee.

*Chemicals*

PTZ,arachidonyl-2-chloroethylamide (ACEA) and paxilline were purchased from Sigma-Aldrich (St. Louis, USA). ACEA was dissolved in a solution containing dimethylsulfoxide (DMSO), chromophore and saline (1:1:18). PTZ was dissolved in distilled water. Paxilline was dissolved in a solution consisting of DMSO and phosphate buffered saline (PBS; 1:10). All the solutions were fresh made at the day of experiment.

Paxilline and ACEA or their vehicles were administered by intraperitoneal (i.p.) injection at the volume of 10 mg/Kg. In all experiments, paxilline or its vehicle was injected 5 min before injection of ACEA or its vehicle. The seizure test was performed 30 min after last drug injection. 

*Maximal electroshock seizure (MES)*

MES is considered as an experimental model allowing to detect the anticonvulsant action of drugs that are clinically used in suppression of tonic–clonic seizures and partial convulsions with or without secondary generalization in humans (16, 17). An alternating current of 50 Hz and 35 mA was delivered to animals (N = 9-12 in each group) through ear-electrodes for 0.2 s. Electrodes were moistened by saline before attaching the ear of the mouse to improve electrical contact. Prevention of MES-induced hindlimb tonic extension (HLTE) was considered as protection against MES-induced seizure.

*Pentylenetetrazole (PTZ)- Induced Seizure*

PTZ-induced seizures model is thought to be an animal model of myoclonic convulsions and, to a certain extent, of absence seizures in humans (18). Groups of mice (N = 10) were randomly selected. Seizure was induced by intravenous (i.v.) infusion of 1% PTZ solution at the constant rate of 0.25 mL/min using an infusion pump (model 53140, Stoelting Inc., USA) through tail vein. A 30-gauge needle with flexible tube, allows infusion of PTZ to unrestrained animal. Infusion was stopped either when a tonic-clonic convulsion with loss of righting reflex was achieved or after the animal received a maximum amount of 1 mL (10 mg) PTZ. The dose of PTZ (mg/Kg) administered to induce tonic-clonic seizure with loss of righting reflex was considered as seizure threshold.

*Statistical analysis*

Data were analyzed by SPSS 16 (SPSS Inc., 2007). In PTZ model, the threshold dose to induce generalized tonic-clonic seizure with loss of writing reflex was compared between experimental groups using Kruskal-Wallis test followed by Dunn’s post hoc test. In MES model, in order to determine the type of interaction between cannabinoid compounds and paxilline, the logistic regression was used to relate the proportion of experimental subjects responding in a predetermined way to the various levels of the drugs given in combination. For a combination of two drugs, the model used is of the following form:

Log[*p*(1-*p*)]= _0_ + β_1_x_1_ + β_2_x_2_ + β_12_x_1_x_2_

where *p* is the proportion of animals responding, x1 represents dose of ACEA (mg/Kg), x2 represents dose of paxilline (mg/Kg). β_0_ is unknown parameter associated with the background response rate. β_1_ is unknown parameter associated with the effect of ACEA. β2 is unknown parameter associated with the effect of paxilline. Also β_12_ is unknown parameter associated with the interaction between ACEA and paxilline. The *p* values less than 0.05 were considered to be statistically significant.

## Results

*The effect of ACEA and paxilline in acute PTZ-Induced seizure threshold*

The results were shown in Table 1. A significant change in seizure threshold was seen among groups (p < 0.0001). Further analysis by Dunn’s test revealed that pretreatment of mice with ACEA increased seizure threshold which was significant at the dose of 5 (p < 0.01) and 10 (p < 0.001) mg/Kg compared with the control group. Although pretreatment with paxilline (1 mg/Kg) *per se* did not changed seizure threshold; however, co-administration of paxilline and ACEA attenuated the protective effect of ACEA at the dose of 5 mg/kg. Co-administration of paxilline (10 mg/Kg, but not 1 mg/Kg) and ACEA (10 mg/Kg) inhibited the protective effects of ACEA against PTZ-induced seizure.

*The effect of ACEA and paxilline in acute MES model of seizure*

The results were shown in Table 2. The control group which received vehicle showed 10% protection against electroshock-induced seizure. Administration of ACEA at the doses of 0.5, 1 5, 10 and 50 mg/Kg resulted in 10%, 10%, 30%, 40%, and 85% protection against MES, respectively. Statistical analysis revealed a significant protective effect of ACEA per se against MES-induced seizure (p = 0.003, Table 3). Administration of paxilline per se at the doses of 0.5, 1, 5, 10, and 50 mg/Kg produced 10%, 30%, 40%, 25% and 50% protection, respectively. The main effect of paxilline in protection against MES-induced seizure was not statistically significant (p = 0.155, Table 3). Overall, co-administration of different doses of paxilline and ACEA produced antagonistic interaction against MES-induced seizure (p = 0.032, Table 3). Interestingly, co-administration of paxilline (0.5 mg/Kg) and ACEA (0.5 mg/Kg) produced a synergistic interaction in protection against MES-induced seizure. 

## Discussion

Our results showed that i.p. administration of ACEA *per se* (at the doses of 5 and 10 mg/Kg) produce a protective effect against PTZ-induced seizure. Moreover, according to estimated value from logistic regression analysis of MES data, it could be suggested that ACEA also has a protective effect against MES-induced seizure (the *p*-value associated with ACEA effect was 0.003; Table 3). These findings are consistent with the results of previous studies suggesting antiepileptic activities of ACEA in these two models of seizure (19-21). CB1 receptors play an important role in inhibition of the neuronal excitability caused by activation of glutamate NMDA receptors. NMDA receptor activity triggers generation of endogenous cannabinoids through increasing cytoplasmic calcium. CB1 receptor ligands exert their effect either by decreasing the pre-synaptic release of glutamate or through participation in post-synaptic NMDA receptors signaling pathways (22). Moreover, calcium channels could also be involved in ACEA antiseizure properties, as shown in previous results indicating that ACEA (2.5 mg/Kg, i.p.) significantly increased the antiepileptic effect of pregabalin in the mouse MES-induced seizure model by significant reducing the median effective dose (ED_50_) of pregabalin (23). Other research results imply that the antiepileptic activity of cannabinoid compounds is mediated, at least partially, through L-type Ca^2+^ channels in PTZ-induced and chronic model of electrical kindling seizure in rats. Co-administration of the L-type Ca^2+^ channel blocker verapamil and ACEA prevented the protective effect of the cannabinoid compound against PTZ-induced seizure (24).

**Table 1:** The protective effects of i.p. administration of ACEA and Paxilline alone or in combination against PTZ-induced seizure in mice. PTZ (1%) was infused through tail vain of mice. The dose of PTZ through for induction of tonic-clonic seizure with loss of righting reflex was major. N = 10 for each group

**PTZ(mg/Kg)**	**Paxilline(mg/Kg)**	**ACEA(mg/Kg)**
18.00 ± 2.69	0	0
23.87 ± 2.89	0	0.5
27.94 ± 5.25	0	1
37.34 ± 3.44 **	0	5
78.06 ± 4.31 ***	0	10
17.70 ± 1.22	1	0
17.82 ± 3.36	1	1
26.90 ± 2.62	1	5
49.07 ± 9.91 **	1	10
20.33 ± 2.88	10	10

* p < 0.05,

*** p < 0.001 significant difference compared to control group [ACEA 0 (mg/Kg) +Paxilline 0(mg/Kg)].

**Table 2:** The protective effects of i.p. administration of ACEA and paxilline alone or in combination against electroshock-induced seizure in mice. The number of mice did not show hind-limb extension in each group was considered as percent protection. N = 9-12 for each group

**Seizure protection **	**Paxilline (mg/Kg)**	**ACEA (mg/Kg)**
10%	0	0
10%	0	0.5
10%	0	1
30%	0	5
40%	0	10
85%	0	50
10%	0.5	0
30%	1	0
40%	5	0
25%	10	0
50%	50	0
60%	5	5
66%	5	1
80%	10	1
66%	10	5
90%	0.5	0.5
40%	1	1
60%	5	0.5

**Table 3:** Estimates of the model parameters and the P values associated with their test of significance for ACEA and paxilline. Data are analyzed using logistic regression method

**Significance**	**S.E.**	**Estimate**	**Parameter**
0.003	0.015	-.046	β_1_(ACEA)
0.155	0.014	-.019	β_2_ (PAXILLINE)
0.032	0.014	-.031	β_12_(interaction ACEA*PAXILLINE)
0.001	0.199	.689	β_0 _(intercept)

Alongside the calcium channels, certain potassium channels are also involved in seizure process, especially those channels that their activation is related to change in intracellular calcium concentrations; namely BK and SK channels. The importance of BK channels in neuronal hyperexcitability and epilepsy formation is due to their unique gating properties; which are affected by both membrane depolarization and rise in intracellular calcium levels. The outward K+ current through BK channels cause a hyperpolarization of the membrane (25). BK channels are widely expressed throughout the central nervous system (CNS) and control neuronal excitability (6). These channels can be activated through an increase in concentration of intracellular Ca^2+^ during the action potential. Therefore, opening of BK channels allows K^+^ to passively flow through the channel, down the electrochemical gradient and contribute to cell repolarization and the fast-afterhyperpolarization (fAHP) which can help set firing rates both at the single-cell and network level (15). The importance of malfunction of BK channels in pathophysiology of seizure comes from the results of previous studies suggesting the involvement of the gene encoding beta regulatory subunit of BK channels in idiopathic generalized seizure (26). A gain-of-function in BK-channel flow has been linked to spontaneous seizures in both animal models and humans. Knockout of the regulatory beta subunit, which normally represses BK-channel currents, leads to spontaneous seizures in mice. Also, seizure itself induces a gain-of-function in BK channels that is associated with increased irritability in neocortical neurons (11, 15, 27). Previous study in our lab showed that BK channel antagonist paxilline have an anticonvulsive effect in PTZ-induced (28) and pilocarpine-induced (12) model of seizures. However, the results of the present study showed that paxilline *per se* had no significant effect (either anticonvulsive or proconvulsive in both PTZ and MES-induced acute seizure) at least at the doses used in this study. Interestingly, pre-treatment of mice with paxilline attenuate the anticonvulsive effects of ACEA in both PTZ and MES models of seizures. The effect of cannabinoid compounds on calcium channels and intracellular calcium gradient can partly explain the antagonistic interaction between ACEA and paxilline. A correlation between neuroprotection and cannabinoids may be via the modulation of intracellular calcium homeostasis to sustain healthy physiological function. Some of the cannabinoids have been proven to regulate calcium homeostasis in the hippocampus (29). 

Mechanisms by which cannabinoids may affect calcium homeostasis consist of the regulation of NMDA receptor stimulation (30, 31), restrain of voltage gated calcium channels (32, 33), potassium channels (34, 35), and gap junction modulation (36). Jin *et al.* showed that inhibition of BK channels could inhibit epileptiform activity in acute seizure models, followed by an increase in intracellular calcium released from intracellular sources (37). 

The different action of ACEA and paxilline on intracellular calcium levels during repetitive firing could explain in part their antagonistic interaction in animal models of seizure. In addition, it was shown that under certain conditions, cannabinoids activate BK channels. Indeed, some unknown factors in the cytoplasm mediate the capability of endogenous cannabinoids to activate BK channel currents. Cannabinoids may be hyperpolarizing factors in cells, such as arterial myocytes, wherein BK channels are highly expressed (38).

To conclude, it could be suggested that despite a protective effect of BK channel antagonists against seizure, pretreatment of mice with paxilline immediately before CB1 receptor agonist administration could diminish the protective effect of the CB1 receptor agonist. The overlap between cannabinoid pharmacologic actions and BK channels activation is the cytoplasmic calcium concentration and the interactions between these two receptors system might be due to their different actions on intracellular calcium levels, suggesting that the antiepileptic activity of ACEA is partially due to the reduce in intracellular calcium levels that is probably mediated by BK channels during seizure.
